# Platelet Function During Extracorporeal Membrane Oxygenation in Adult Patients: A Systematic Review

**DOI:** 10.3389/fcvm.2018.00157

**Published:** 2018-11-09

**Authors:** Camilla Mains Balle, Anni Nørgaard Jeppesen, Steffen Christensen, Anne-Mette Hvas

**Affiliations:** ^1^Department of Clinical Biochemistry, Aarhus University Hospital, Aarhus, Denmark; ^2^Department of Anesthesiology and Intensive Care Medicine, Aarhus University Hospital, Aarhus, Denmark; ^3^Department of Clinical Medicine, Aarhus University, Aarhus, Denmark

**Keywords:** blood platelets, extracorporeal membrane oxygenation, extracorporeal life support, platelet activation, platelet aggregation, platelet function tests, systematic review

## Abstract

**Background:** Hemorrhagic and thromboembolic complications are common during treatment with extracorporeal membrane oxygenation (ECMO), resulting in considerable morbidity and mortality. This emphasizes the clinical relevance of understanding hemostatic changes occurring during ECMO treatment. As platelets are key players in hemostasis, detailed knowledge on how ECMO treatment affects platelet function is of great importance. We therefore aimed to systematically summarize and discuss existing knowledge on platelet function during ECMO treatment in adult patients.

**Methods:** Systematic review complying with the Preferred Reporting Items for Systematic Reviews and Meta-Analyses (PRISMA) guidelines. Objectives and methods were specified in a PROSPERO protocol (ID no CRD42018084059). The MEDLINE/PubMed, EMBASE, and Web of Science databases were systematically searched on September 10, 2018. A standardized quality assessment tool was used to assess the risk of bias in included studies. Primary outcome was platelet function during ECMO treatment, measured as platelet adhesion, activation or aggregation. Secondary outcomes were thrombosis, bleeding, and mortality during ECMO treatment.

**Results:** A total of 591 studies were identified, of which seven were eligible for inclusion in the qualitative synthesis. Of these, one study investigated expression of platelet adhesion receptors and found them to be reduced during ECMO treatment; two studies reported a decrease in platelet activation markers during ECMO treatment; and five studies demonstrated reduced platelet aggregation during ECMO treatment. Three studies reported on thrombosis, mortality and/or bleeding during ECMO treatment; no thromboembolic events were reported; all three studies reported frequent bleeding episodes defined on basis of transfusion requirements. An in-hospital mortality of 35–40% and a 30-day mortality of roughly 30% were reported in three different studies.

**Conclusions:** The present systematic review reveals a substantial knowledge gap regarding platelet function during ECMO treatment in adult patients and underscores the demand for more and well-designed studies on this topic. There is suggested evidence of reduced platelet adhesion, decreased platelet activation, and reduced platelet aggregation in adult patients during ECMO treatment. Importantly, platelet aggregation results need to be interpreted in the light of low platelet counts. The associations of platelet function and bleeding and/or thromboembolic complications during ECMO treatment remain to be fully elucidated.

## Introduction

Extracorporeal membrane oxygenation (ECMO) can be lifesaving in patients with respiratory and/or cardiac failure refractory to conventional treatment. In recent years, the use of ECMO has increased dramatically (1), most likely owing to studies reporting favorable outcomes for adult ECMO patients (2–4) in combination with advances in technology.

Hemorrhagic and thromboembolic complications are common during ECMO treatment, resulting in considerable morbidity and mortality (5–8). Bleeding is the most common complication, occurring in 29–33% of adult ECMO patients (7, 9). Intracranial hemorrhage, the most potentially devastating bleeding complication, is reported to occur in around 5% of adult ECMO patients (5, 6). Thromboembolic complications are generally less common; intracranial infarction is reported to occur in 2–5% (5, 6) and venous thrombosis in 10% of adult ECMO patients (9). Although some thromboembolic events are rapidly clinically apparent, many are subclinical and the true incidence of thromboembolic complications during ECMO is therefore likely underestimated (10). In addition, clot formation in the circuit can lead to oxygenator failure and exchange of the entire circuit, which is reported to occur in up to nearly one third of adult ECMO runs (5, 9).

Several mechanisms that could affect platelet function during ECMO treatment have been investigated *in vitro*, suggesting that platelets become both activated *and* impaired during ECMO treatment. Artificial surfaces, such as the extracorporeal circuit, induce platelet adhesion and activation (11). Furthermore, high shear stress, as in the ECMO circuit, has been shown to cause enhanced platelet activation (12, 13), which may lead to an increased thrombotic propensity. On the contrary, high shear stress has also been shown to cause loss of platelet surface receptors important for platelet adhesion (12, 13), as well as loss of high-molecular-weight von Willebrand factor multimers, resulting in decreased binding of von Willebrand factor to platelets (13, 14). Both mechanisms may result in impaired platelet adhesion and hence impaired activation, leading to an increased risk of bleeding. The risk of bleeding is further augmented by ongoing consumption of platelets due to platelet adhesion to the surface of the circuit, and formation of microthrombi in the circulation.

The increased thrombotic propensity necessitates antithrombotic therapy to conserve patency of the ECMO circuit and to reduce thrombotic complications. Although anticoagulation guidelines vary widely among ECMO centers, unfractionated heparin (UFH) is the most widely administered anticoagulant (15). The use of UFH can, however, result in bleeding, posing an additional risk to the patient.

Limited data exist on platelet function during ECMO treatment in adult patients (16–22) and no systematic review has yet summarized the current knowledge. Consequently, the aim of the present systematic review was to summarize and discuss existing knowledge regarding platelet function during ECMO treatment in adult patients.

## Methods

The present systematic review complies with the Preferred Reporting Items for Systematic Reviews and Meta-Analyses (PRISMA) guidelines (23). Prior to data extraction, the objectives and methods were specified in a PROSPERO protocol (ID no. CRD42018084059).

### Search strategy

The MEDLINE/PubMed, Embase, and Web of Science databases were systematically searched for relevant publications on September 10, 2018, without time boundaries. All searches were filtered to include English language only. A manual search of the references identified relevant articles supplementing the electronic search. Search terms were as follows: ***PubMed:*** (((((((extracorporeal membrane oxygenation (24)) OR extracorporeal life support) OR extracorporeal cardiopulmonary resuscitation) OR ecmo) OR ecls) OR ecpr)) AND ((((((((platelet activation [MeSH]) OR platelet aggregation [MeSH]) OR platelet activity) OR platelet function) OR platelet dysfunction) OR platelet function tests [MeSH]) OR flow cytometry [MeSH]) OR aggregometry). ***Embase:*** (“extracorporeal membrane oxygenation”/exp OR“extracorporeal membrane oxygenation” OR “ecmo” OR “extracorporeal life support” OR “ecls” OR “extracorporeal cardiopulmonary resuscitation” OR “ecpr” OR “extracorporeal oxygenation”) AND (“thrombocyte activation”/exp OR “platelet activation” OR “thrombocyte activity” OR “platelet activity” OR “thrombocyte aggregation”/exp OR “platelet aggregation” OR “thrombocyte function”/exp OR “platelet function” OR “thrombocyte dysfunction”/exp OR “platelet dysfunction” OR “thrombocyte function analyzer”/exp OR “flow cytometry”/exp OR “flow cytometry” OR “aggregometry”) AND [English]/lim. ***Web of Science:*** (extracorporeal membrane oxygenation OR extracorporeal life support OR extracorporeal cardiopulmonary resuscitation) AND (platelet activ^*^ OR thrombocyte activ^*^ OR platelet function OR thrombocyte function OR platelet dysfunction^*^ OR thrombocyte dysfunction^*^ OR platelet aggregation OR thrombocyte aggregation OR platelet function test^*^ OR thrombocyte function test^*^ OR flow cytometry OR aggregometry) AND language: (English).

### Study selection

Fifty abstracts were randomly chosen and screened independently by two authors (CB, AH). The abstracts were screened based on predefined in- and exclusion criteria and any divergences were discussed to reach consensus. One author (CB) screened the remaining 541 abstracts. Studies eligible for full-text reading were assessed by two authors (CB, AH). Publications were included if they met the following criteria: (a) original data; (b) adult patients (≥18 years) receiving ECMO treatment; (c) platelet function determined (platelet adhesion, platelet activation, or platelet aggregation); (d) English language. Exclusion criteria were: (a) reviews or guidelines; (b) letters, editorials or comments without original data; (c) case reports with <5 cases; (d) *in vitro* or animal data; (e) posters or conference abstracts; (f) only platelet count determined.

### Data extraction

Data extraction was performed by CB and verified by AH. Platelet function analyses were categorized as measurements of platelet adhesion, activation, or aggregation. Platelet adhesion analyses were defined as measurements of adherence to foreign surfaces *or* measurements of adhesion potential (expression of surface receptors essential to platelet adhesion); platelet activation analyses as measurements of soluble markers of platelet activation *or* platelet activation potential (expression of activation-dependent platelet surface markers); and platelet aggregation analyses as functional analyses measuring platelet aggregation.

To assess the potential risk of bias in the included studies, two authors (CB, AH) independently evaluated each study using a standardized study quality assessment tool from the National Heart, Lung, and Blood Institute, US (25). Any discrepancies were discussed to reach consensus.

## Results

**Figure 1:**
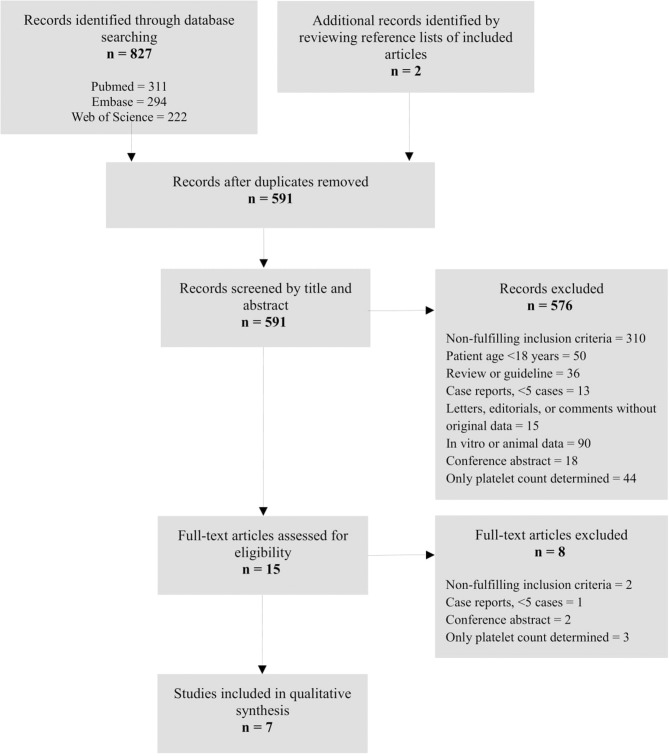
Flow chart depicting the systematic literature search and the study selection process.

The systematic literature search returned a total of 591 studies. In total, 584 studies were excluded, resulting in seven studies to be included in the qualitative synthesis. Figure 1, modified from (23), shows a flow chart outlining the screening and selection process. During the process of full-text screening, the study by Nair et al. was included in the final synthesis despite their inclusion criterion of age >16 years (21). The article contained no information specifying whether any patients aged <18 years were included, and data could not be obtained from the investigators. However, the median age of included patients was 41 years [interquartile range (IQR): 38–52 years] indicating that very few, if any, patients were below 18 years of age.

A summary of included studies is presented in Table 1. All seven studies were observational cohort studies. Using the study quality assessment tool, the study by Tauber et al. rated *good* (19), five studies rated *fair* (16–18, 20, 21), and one study rated *poor* (22).

**Table 1:** Studies included in the systematic review investigating platelet function during extracorporeal membrane oxygenation in adult patients.

**Year, Author, Quality rating**	**Patients, n ECMO configuration ECMO duration ECMO indication**	**Antithrombotic therapy**	**Markers of platelet function (method)**	**Results**		
				**Platelet count × 10^9^/L, mean ± SD**	**Platelet function**	**Clinical events (definition)**
**PLATELET ADHESION**						
Lukito et al. (16)*Rating*fair	*n* = 20 *Configuration* 14 VA + 6 VV *Duration* Median: 4 days (range 1–11) *Indication* Cardiomyopathy (*n* = 9) Respiratory failure (*n* = 7) Cardiac arrest (*n* = 2) Others (*n* = 2)	VKA + ASA + dipyridamole (*n* = 1) VKA + ASA (*n* = 1) VKA only (*n* = 1) Heparin^#^ + antiplatelets (*n* = 3) Heparin^#^ only (*n* = 9) No antithrombotics (*n* = 5)	Soluble GPVI (ELISA) Surface-bound GPIbα and GPVI (flow cytometry) Integrin α_IIb_ subunit (flow cytometry)	*Unspecified time point during ECMO:* 132 (range 51–268)	*Unspecified time point during ECMO compared with healthy individuals:* ↓ GPIbα ↓ GPVI ↑ sGPVI → Integrin α_IIb_ subunit	*Thrombosis* Not reported *Bleeding* Not reported *Mortality* Not reported
**PLATELET ACTIVATION**						
Chung et al. (17)*Rating*Fair	*n* = 13 *Configuration* VV *Duration* Median: 7 days (range 6–7) *Indication* Acute respiratory failure (*n* = 10) Acute-on-chronic lung disease (*n* = 2) Pulmonary embolism (*n* = 1)	UFH Target ACT = 140–160 s.	β-thromboglobulin (ELISA) Platelet factor 4 (ELISA)	*During ECMO compared with before ECMO:* Widely diverging platelet counts – no significant trend over time. *At ECMO cannulation:* 194 ± 74	*72 h. on ECMO compared with before ECMO:* ↓β-thromboglobulin (↓) Platelet factor 4	*Thrombosis* Not reported *Bleeding* Not reported *Mortality* Not reported
**PLATELET AGGREGATION**						
Laine et al. (18)*Rating*Fair	*n* = 23 *Configuration* 18 VA + 5 VV *Duration* Range 3–30 days *Indication* Cardiac surgery (*n* = 6) Dilated cardiomyopathy (*n* = 2) Acute myocardial infarction (*n* = 2) Cardiac failure/cardiogenic shock (not AMI) (*n* = 1) Myocarditis (*n* = 2) Respiratory failure (*n* = 3) Pulmonary artery hypertension (*n* = 1) Pneumonia (*n* = 3) Complications of pulmonary/cardiac transplantation (*n* = 3)	UFH Target ACT: PLC > 100 × 10^9^ /L: 180–200 s. PLC < 100 × 10^9^ /L: 160–180 s.	Platelet aggregation (Multiplate Analyzer, ADP, TRAP, AA as agonists) *Agonist concentrations* Not reported	*Before ECMO*, *median:* 130 *During ECMO compared with before ECMO:* ↓^ϕ^ PLC	*Before and during ECMO compared with healthy individuals:* ↓^ϕ^ Platelet aggregation (TRAP, AA, ADP) *During ECMO compared with before ECMO:* ↓^ϕ^ Platelet aggregation (TRAP) ↑^ϕ^ Platelet aggregation (AA) → ^ϕ^ Platelet aggregation (ADP)	*Thrombosis* No events (A venous or arterial thromboembolism leading to neurological complication or pulmonary embolism) *Bleeding* Severe bleeding: 26.1% (*n* = 6) (Two or more liters in the first 24 h following cannulation or administration of more than five units of RBC in 24 h) *Mortality* 30-day mortality: 30.4%
Tauber et al. (19)*Rating*Good	*n =* 38 *Configuration* 26 VA + 12 VV *Duration* Not reported *Indication* Intractable cardiac or respiratory failure (*n =* 38)	UFH Target ACT = 150–180 s.	Platelet aggregation (Multiplate Analyzer, ADP 6.4 μM, TRAP 32 μM, AA 0.5 mM as agonists)	*Before ECMO:* 134 ± 95 *During ECMO compared with before ECMO:* ↓ PLC *During ECMO compared with after ECMO:* ↓ PLC *Before ECMO compared with after ECMO:* → PLC	*Before ECMO compared with healthy individuals:* ↓ Platelet aggregation (ADP, AA) → Platelet aggregation (TRAP) *24 h. on ECMO compared with before ECMO:* ↓ Platelet aggregation (ADP, AA, TRAP) *48 h. on ECMO compared with before ECMO:* ↓ Platelet aggregation (ADP, AA) **(**↓**)** Platelet aggregation (TRAP) *24 h. after ECMO compared with before ECMO:* → Platelet aggregation (TRAP, ADP, AA)	*Thrombosis* Not reported *Bleeding* Moderate (< 2U RBC/d) *n =* 28 (74%) Increased (≥ 2U RBC/d): *n =* 10 (26%) (Estimated indirectly by transfusion requirements) *Mortality* Not reported
Mutlak et al. (20)*Rating*Fair	*n =* 5 *Configuration* VV *Duration* Not reported *Indication* ARDS (*n =* 5)	UFH Target aPTT = 45–55 s.	Platelet aggregation (Multiplate Analyzer, ADP 0.4 mM, TRAP 32 mM, AA 0.5 mM as agonists)	*Before ECMO:* 136 ± 30	*90 min. on ECMO compared with before ECMO:* ↓ Platelet aggregation (ADP) (↓) Platelet aggregation (AA) → Platelet aggregation (TRAP) *120, 150 and 180 min. on ECMO compared with before ECMO:* ↓ Platelet aggregation (ADP) (↓) Platelet aggregation (AA) → Platelet aggregation (TRAP)	*Thrombosis* Not reported *Bleeding* Not reported *Mortality* Not reported
Nair et al. (21)*Rating*Fair	*n =* 10 *Configuration* 7 VA + 3 VV *Duration* Median: 10 days (IQR 5–14) *Indication* Primary graft dysfunction (lung) (*n =* 2) Severe ARDS (*n =* 1) Primary graft dysfunction (heart) (*n =* 3) Cardiogenic shock (*n =* 2) Post cardiotomy (*n =* 2)	UFH Target aPTT = 1.5–2 x normal value	Platelet aggregation (Multiplate Analyzer, ADP, TRAP, collagen, ristocetin as agonists) *Agonist concentrations* Not reported	*During ECMO compared with before ECMO:* ↓^ϕ^ PLC	*Unspecified time point during ECMO compared with healthy individuals:* ↓^ϕ^ Platelet aggregation (ADP, TRAP, collagen, ristocetin)	*Thrombosis* No events (Clinically apparent arterial or venous thrombosis or oxygenator failure) *Bleeding* 5 (50%) experienced bleeding episodes. (Any bleeding significant enough to trigger administration of blood products (excluding RBC) or coagulation factors) *Mortality* In-hospital mortality: 40%
**PLATELET ACTIVATION AND AGGREGATION**						
Kalbhenn et al. (22)*Rating*Poor	*n =* 6 (flow cytometry) *n =* Not reported (aggregometry) *Configuration* VV *Duration* Mean: 10 days (SD 9.5) *Indication* ARDS Acute interstitial pneumonia Exacerbated COPD Bridge to lung transplant Post-lung transplant Post-pneumectomy Tracheal rupture	UFH Target aPTT = 40 s. *or* anti-factor Xa activity ≤ 0.2 U/ml LMWH Target anti-factor Xa activity ≤ 0.18 IU/ml	Surface-bound CD62 and CD63 (flow cytometry) Platelet aggregation (light transmission aggregometry, collagen, ristocetin, ADP, epinephrin as agonists) *Agonist concentrations* Not reported	*During ECMO compared with before ECMO:* ↓ PLC *During ECMO compared with after ECMO:* ↓^ϕ^ PLC *Before ECMO compared with after ECMO:* → PLC	*On ECMO compared with healthy individuals:* ↓CD62 ↓CD63 *On ECMO compared with after ECMO:* ↓Platelet aggregation (ADP, ristocetin) (↓) Platelet aggregation (collagen, epinephrine)	*Thrombosis* Not reported *Bleeding* Not reported *Mortality* In-hospital mortality: 35%

*↑ Statistically significant increase; ↓ Statistically significant reduction; → No change; (↑) Non-significant increase; (↓) Non-significant reduction*;

ϕ *Significance not tested*;

# *Type of heparin not specified*.

*ECMO, Extracorporeal membrane oxygenation; VV, Veno-venous; VA, Veno-arterial; ARDS, Acute respiratory distress syndrome; COPD, Chronic obstructive pulmonary disease; ACT, Activated clotting time; aPTT, Activated partial thromboplastin time; UFH, Unfractionated heparin; LMWH, Low molecular weight heparin; VKA, Vitamin K antagonist; ASA, Acetylsalicylic acid; ELISA, Enzyme-linked immunosorbent assay; GPIV, Glycoprotein VI; GPIb, Glycoprotein Ib; TRAP, Thrombin receptor-activating peptide-6; ADP, Adenosine diphosphate; AA, Arachidonic acid; PLC, Platelet count; RBC, Red blood cells; AUC, Area under the curve; IQR, Interquartile range*.

### Platelet count

Five studies reported platelet counts at several time points during the study period. Of these, two studies measured platelet counts prior to and during ECMO treatment (18, 21); they reported lower platelet counts during ECMO treatment compared with before ECMO initiation, and persistently lower platelet counts during ECMO treatment compared with healthy individuals. Two studies measured platelet counts both prior to, during, and after ECMO treatment (19, 22); they demonstrated significantly lower platelet counts during ECMO treatment compared with before ECMO initiation; lower platelet counts during ECMO compared with healthy individuals; and after termination of ECMO, platelet counts did not differ from platelet counts before ECMO initiation. In contrast, Chung et al. reported widely diverging platelet counts during the course of ECMO treatment, demonstrating no uniform changes in platelet counts over time (17). The remaining two studies measured platelet counts at a single time point during the study period (16, 20); Mutlak et al. measured platelet counts prior to ECMO initiation and found them to be reduced compared with healthy individuals (20); similarly, Lukito et al. measured platelet counts at an unspecified time point during ECMO treatment and found the counts to be reduced compared with healthy individuals (16). The effects of administered platelet transfusions on platelet counts were not accounted for in any of the included studies.

### Platelet function

Lukito et al. reported reduced levels of adhesion receptors for collagen and vWF (GPVI and GPIbα) on circulating platelets compared with healthy individuals, at a single, unspecified time point during ECMO treatment (16); furthermore, they reported an increase in the level of soluble GPVI in ECMO patients.

Chung et al. investigated platelet function by measuring soluble markers of platelet activation (17); they reported a decrease in platelet-activation markers, β-thromboglobulin and platelet factor 4, during the first 72 h of ECMO treatment compared with before ECMO initiation. On day seven of ECMO treatment, β-thromboglobulin levels remained low, while platelet factor 4 levels had returned to a level corresponding to before ECMO initiation. Kalbhenn et al. investigated platelet activation by measuring the expression of activation-dependent platelet surface markers: CD62 (P-selectin) and CD63; they reported reduced surface expression of CD62 and CD63 compared with healthy individuals, at a single, unspecified time point during ECMO treatment.

Five studies investigated platelet function by measuring platelet aggregation. Four studies employed whole blood impedance aggregometry (18–21) and demonstrated reduced platelet aggregation during ECMO treatment compared with either before ECMO initiation (18–20) or with healthy individuals (21). Tauber et al. (19) also measured platelet aggregation after ECMO termination and found no difference in platelet aggregation 24 h after ECMO compared with before ECMO initiation. Two studies demonstrated reduced platelet aggregation compared with healthy individuals even before initiation of ECMO (18, 19). Kalbhenn et al. employed light transmission aggregometry when measuring platelet aggregation at two unspecified timepoints during and after ECMO treatment (22). They excluded all patients with platelet counts <100 × 10^9^ /L from platelet aggregation analyses. They reported a decrease in platelet aggregation (for ADP and ristocetin) or no difference in platelet aggregation (for collagen and epinephrine) during ECMO compared with after ECMO treatment.

### Clinical events

Four out of seven studies reported major clinical events: thrombosis, bleeding, and mortality (18, 21); bleeding alone (19); or mortality alone (22). No thromboembolic events were reported during ECMO treatment (18, 21). Patients frequently required transfusions of blood products; Laine et al. reported severe bleeding in 26% of patients (18) from either cannula insertion sites or postoperative chest tube drains; Nair et al. reported bleeding episodes in 50% of patients without reporting bleeding sites or severity (21); and Tauber et al. reported transfusion requirements of ≥2 units of red blood cells (RBC) per day in 26% of patients without reporting bleeding sites (19). In addition, Tauber et al. (19) found an increased transfusion requirement (≥2 units RBC/day) during ECMO to be associated with lower platelet aggregation values [using thrombin receptor activating peptide-6 (TRAP) as agonist] compared with a moderate transfusion requirement (<2 units RBC/day). In-hospital mortalities of 35% (22) and 40% (21), and a 30-day mortality of 30.4% (18) were reported.

## Discussion

This systematic review revealed that only a limited number of published studies investigate platelet function in patients during ECMO treatment; only seven studies investigating platelet function during ECMO treatment in adult patients were identified. They suggest evidence of a reduced potential for platelet adhesion, and decreased platelet activation during ECMO treatment. Furthermore, platelet aggregation is reduced during ECMO treatment compared with either before ECMO initiation, after ECMO termination, or with healthy individuals. The association between platelet function and bleeding/thromboembolic complications during ECMO treatment remains to be fully elucidated.

All included studies were rated using a study quality assessment tool (25). Tauber et al. (19) rated *good*, suggesting a low risk of bias, whereas *fair* ratings of five studies (16–18, 20, 21) insinuated a greater risk of bias of the estimates. Fair ratings were given due to small cohort sizes with no sample size justification and no *a priori* sample size estimate; retrospective data collection; and/or no statistical adjustment of estimates for potential confounding variables (e.g., platelet count). Justifying a *fair* rating of these studies, however, were detailed and structured descriptions of exposure and outcome measures together with transparent presentations of data. One study (22) was rated *poor*, due to insufficient information regarding the study population and methods employed.

Lukito et al. reported significant reductions in the expression of adhesion receptors (GPIbα and GPVI) on circulating platelets in ECMO patients compared with healthy individuals (16). Loss of the GPIbα and GPVI receptors reduces the binding capacity of platelets to vWF and collagen, leading to impaired platelet adhesion and hence impaired platelet activation (26, 27). Recently, Chen et al. supported these findings by demonstrating loss of platelet receptors, GPIbα and GPVI, during *in vitro* conditions of high shear stress (12). The loss of receptors resulted in reduced adhesion of platelets to collagen and vWF (12). However, Chen et al. also measured adherence of the sheared platelets to fibrinogen, deposited on an artificial surface, demonstrating that high shear stress also induces platelet activation, thereby increasing thrombotic propensity (12). The authors proposed that these two concurrent mechanisms, leading to impaired *and* enhanced platelet activation, may explain why bleeding and thrombotic complications occur simultaneously in patients treated with ECMO (12).

The increase in soluble GPVI levels, measured by Lukito et al., indicates proteolytic shedding as the main mechanism for reduced adhesion-receptor levels (16). Their findings are supported by Al-Tamimi et al., who reported metalloproteinase-dependent shedding of surface-bound GPVI after exposure of platelets to high shear stress *in vitro* (28). In line with others, they proposed that this shear-induced GPVI shedding may be a protective mechanism for down-regulating platelet adhesiveness during elevated shear stress, thereby reducing the level of platelet activation and thrombus formation (28, 29). Shedding of platelet adhesion receptors has been shown to accompany platelet activation (30, 31). Therefore, reduced levels of GPIb and GPVI measured by Lukito et al. may be suggestive of enhanced platelet activation during ECMO treatment (16).

In contrast, Chung et al. reported reduced platelet activation during ECMO treatment, demonstrated by decreasing levels of plasma β-thromboglobulin and platelet factor 4 during the first 72 h on ECMO treatment compared with before ECMO initiation (17). This may be explained by two distinct mechanisms. β-thromboglobulin and platelet factor 4 are thought to be sensitive markers of platelet activation, as they are secreted from the α-granules upon platelet activation (32). Platelets are able to release 100% of their total α-granule content (33). It is therefore plausible that initial extensive or continuous platelet activation would lead to release of the entire β-thromboglobulin and platelet factor 4 content, resulting in exhausted platelets and a decrease in plasma β-thromboglobulin and platelet factor 4 levels. Chung et al. obtained the first post-cannulation blood sample at 24 h after ECMO initiation (17), and an initial rise in β-thromboglobulin and platelet factor 4 would therefore not have been detected due to fast clearance times; the half-life of β-thromboglobulin is reported to be 100 min and platelet factor 4 clearance is even faster (34). The other mechanism to be considered is adherence of β-thromboglobulin to the heparin-coated surface of the ECMO circuit. A recent study by Sagedal et al. (35) suggested absorption of β-thromboglobulin to heparin-coated surfaces, as both β-thromboglobulin and platelet factor 4 are heparin-binding proteins (32). If so, β-thromboglobulin might not be a reliable marker of platelet activation in heparin-coated medical devices including ECMO circuits.

Kalbhenn et al. (22) performed flow-cytometric analyses on platelet rich plasma revealing significantly reduced expressions of CD62 (impaired α-granule secretion) and CD63 (impaired δ-granule secretion) in ECMO patients compared with healthy individuals. Their results are suggestive of a reduced platelet activation potential during ECMO treatment. However, it is interesting to speculate whether the mechanism leading to impaired platelet activation *ex vivo* could be vast activation of platelets *in vivo* during ECMO treatment, resulting in impaired platelet reactivity to agonist stimulation *ex vivo*.

Four out of five studies investigating platelet aggregation employed whole blood impedance aggregometry. All four studies indicated reduced platelet aggregation in adult patients during ECMO treatment (18–21). An inherent limitation of this analysis is a strong association between platelet count and platelet aggregation both within and below the reference interval for platelet count (36–41). Nair et al. (21) excluded data on platelet aggregation from analysis if platelet counts were below 100 × 10^9^/L. Tauber et al. (19) also considered the influence of thrombocytopenia on impedance aggregometry results as a limitation to the study but deemed the effects to be minimal. Employing light transmission aggregometry, Kalbhenn et al. (22) demonstrated reduced platelet aggregation during ECMO treatment (for two out of four agonists) compared with after ECMO termination. They excluded patients from analysis if platelet counts were below 100 × 10^9^ /L; this threshold might be set a little too low, as light transmission aggregometry is influenced by platelet counts below 150 × 10^9^ /L (37). Thus, data from the included studies do not enable us to make firm conclusions regarding platelet aggregation during ECMO treatment, as they might very well be influenced by low platelet counts.

For analyses of platelet aggregation, the most widely used agonists were adenosine diphosphate (ADP), arachidonic acid (AA), and TRAP; only two studies provided information regarding concentrations used (19, 20). Agonist concentrations differed; both studies, however, compared platelet aggregation during ECMO treatment to before ECMO initiation, abating the need for comparable agonist concentrations between studies.

Whether the observed alterations in platelet count and function are caused by the ECMO circuit itself or rather the underlying disease is difficult to determine. However, two studies clearly demonstrated significantly reduced platelet counts during ECMO treatment compared with before ECMO initiation and, importantly, they demonstrated that platelet counts recovered to pre-ECMO values within 24 h after ECMO termination (19, 22). This indicates that the reduction in platelet count was caused by the ECMO treatment. With regard to platelet function, there is suggested evidence that ECMO treatment induces platelet dysfunction; three studies demonstrated a decrease in platelet aggregation compared with before ECMO initiation (18–20); Kalbhenn et al. found platelet aggregation to increase after ECMO termination; and Tauber et al. demonstrated that platelet aggregation increased to pre-ECMO values within 24 h after ECMO termination. Additional studies investigating ECMO treatment as a cause of thrombocytopenia and platelet dysfunction are needed to strengthen these proposed associations.

Inflammation has been shown to induce bleeding in thrombocytopenic mice (42). Only one of the included studies provided information regarding inflammatory parameters measured during ECMO treatment (17); furthermore, the associations between bleeding episodes and platelet count or inflammatory parameters were not accounted for in any of the included studies. Laine et al. found no significant associations between platelet aggregation and the risk of bleeding or mortality (18), and no thromboembolic events were reported in any of the included studies; both of which might be attributed to the small cohort sizes. Whether there is an association between platelet function and clinical complications such as bleeding or thromboembolic events therefore remains to be established.

### Strengths and limitations

The systematic approach and a broad search string without time boundaries across three major databases make this systematic review comprehensive, minimizing the risk of overlooking relevant studies. Included studies are thoroughly presented in a transparent manner, including design, quality rating, and results found in each study.

However, some limitations must be considered. Only seven studies compiling with the aim and inclusion criteria were identified. The quality of included studies varied; only one study rated *good* (19), suggesting possible risk of bias in the remaining studies (16–18, 20–22). Furthermore, the patient cohorts were heterogeneous with respect to their underlying diseases (16, 18, 19, 21, 22), which could influence platelet count and function; e.g., patients treated with VV-ECMO, suffering from acute respiratory distress syndrome or severe sepsis, typically have bone marrow suppression, and hence platelet counts drop in these patients independently of platelet consumption in the ECMO circuit. Bone marrow involvement is not typical in the case of patients treated with VA-ECMO, however, these patients are frequently treated with platelet inhibitors, which alter platelet function. Any influences of these differences between ECMO cohorts on the results of this review cannot be assessed based on the data available from the studies included.

The majority of included studies measured platelet function using impedance aggregometry (18–21) entailing the limitation of a strong association between platelet count and platelet aggregation. Recently, our research group reported a model to derive a measure of platelet aggregation adjusted for platelet count (36, 43). This approach could be valuable in evaluating whether platelet aggregation is reduced due to dysfunctional or exhausted platelets, or merely due to a low number of circulating platelets during ECMO treatment.

### Conclusions

The present systematic review reveals a substantial knowledge gap regarding platelet function during ECMO treatment in adult patients and underscores the demand for more and well-designed studies on this topic. At present, there is suggested evidence of reduced platelet adhesion and a decreased platelet activation potential in adult patients during ECMO treatment. Furthermore, platelet aggregation was found to be reduced during ECMO treatment. Importantly, measurements of platelet aggregation were hampered by low platelet counts, why these results need to be interpreted with caution. Whether platelet function is associated with clinical complications such as bleeding and thromboembolic events remains to be established.

## Author contributions

CB and AH performed the systematic literature search, screened all abstracts and full-text articles independently, and performed the study quality assessment of all included articles. CB performed the data extraction, which was verified by AH; CB took the lead in writing the manuscript. All authors (CB, AH, AJ, SC) contributed to the qualitative analysis and provided critical revision of the manuscript.

### Conflict of interest statement

The authors declare that the research was conducted in the absence of any commercial or financial relationships that could be construed as a potential conflict of interest.

## Abbreviations

ECMO: Extracorporeal membrane oxygenation
VV: Veno-venous
VA: Veno-arterial
ARDS: Acute respiratory distress syndrome
COPD: Chronic obstructive pulmonary disease
ACT: Activated clotting time
aPTT: Activated partial thromboplastin time
UFH: Unfractionated heparin
LMWH: Low molecular weight heparin
VKA: Vitamin K antagonist
ASA: Acetylsalicylic acid
ELISA: Enzyme-linked immunosorbent assay
GPIV: Glycoprotein VI
GPIbα: Glycoprotein Ibα
TRAP: Thrombin receptor-activating peptide-6
ADP: Adenosine diphosphate
AA: Arachidonic acid
PLC: Platelet count
RBC: Red blood cells
AUC: Area under the curve
IQR: Interquartile range.
